# Dandelion Root and Lemongrass Extracts Induce Apoptosis, Enhance Chemotherapeutic Efficacy, and Reduce Tumour Xenograft Growth* In Vivo* in Prostate Cancer

**DOI:** 10.1155/2019/2951428

**Published:** 2019-07-17

**Authors:** Christopher Nguyen, Ali Mehaidli, Kiruthika Baskaran, Sahibjot Grewal, Alaina Pupulin, Ivan Ruvinov, Benjamin Scaria, Krishan Parashar, Caleb Vegh, Siyaram Pandey

**Affiliations:** Department of Chemistry and Biochemistry, University of Windsor, Windsor, Ontario, Canada N9B 3P4

## Abstract

Many conventional chemotherapies have indicated side effects due to a lack of treatment specificity and are thus not suitable for long-term usage. Natural health products are well-tolerated and safe for consumption, and some have pharmaceutical uses particularly for their anticancer effects. We have previously reported the anticancer efficacy of dandelion (*Taraxacum officinale*) root and lemongrass (*Cymbopogon citratus*) extracts. However, their efficacy on prostate cancer and their interactions with standard chemotherapeutics have not been studied to determine if they will be suitable for adjuvant therapies. If successful, these extracts could potentially be used in conjunction with chemotherapeutics to minimize the risk of drug-related toxicity and enhance the efficacy of the treatment. We have demonstrated that both dandelion root extract (DRE) and lemongrass extract (LGE) exhibit selective anticancer activity. Importantly, DRE and LGE addition to the chemotherapeutics taxol and mitoxantrone was determined to enhance the induction of apoptosis when compared to individual chemotherapy treatment alone. Further, DRE and LGE were able to significantly reduce the tumour burden in prostate cancer xenograft models when administered orally, while also being well-tolerated. Thus, the implementation of these well-tolerated extracts in adjuvant therapies could be a selective and efficacious approach to prostate cancer treatment.

## 1. Introduction

Prostate cancer remains one of the most commonly diagnosed cancers among men worldwide, accounting for 1 in 5 new diagnoses [1]. Advances in prostate cancer detection including improvement of prostate specific antigen detection methods [2] and functional MRI techniques [3, 4] have resulted in reduced mortality rates due to early diagnosis and treatment of the disease. However, prostate cancer related fatality is associated with late diagnoses and progression to the metastatic castration-resistant stage [5]. At this stage, common treatment approaches involve chemotherapy treatment including first-line therapies such as mitoxantrone and docetaxel [5].

Current treatments for prostate cancer have included chemotherapy, radiation therapy, hormonal therapy, and cryosurgery [6]. Many chemotherapeutic treatment approaches have been developed, targeting vulnerabilities in cancerous cells to induce programmed cell death, also known as apoptosis [7, 8]. Unfortunately, although these treatments have shown efficacy, many adverse side effects have been observed in patients undergoing these treatments [6]. For example, cardiac toxicity has been observed in the usage of mitoxantrone treatment [9, 10]. These side effects can be attributed to the nonspecific nature of the treatment for cancer, resulting in the targeting of healthy noncancerous cells. However, aggressive metastatic cancers pose a constant threat of relapse following remission by chemotherapy treatment. Randomized phase 3 trials have shown that androgen deprivation therapy along with docetaxel improved relapse-free survival in prostate cancer patients [11]; the long-term usage of chemotherapies is highly undesirable due to the inadvertent side effects. Thus, there is a great need for the development of treatment approaches that can avoid treatment-related toxicity and can be used on a long-term basis.

The potential of natural health products (NHPs) in cancer treatments presents an interesting option that can be well-tolerated and could be used over a long period of time. NHPs are materials that have been extracted from plant sources that have exhibited medicinal properties [12]. Interestingly, many of the current chemotherapeutic compounds have been isolated from plant-based materials, for example, paclitaxel (otherwise known as taxol) from the Pacific yew tree (*Taxus brevifolia*) bark extract [13]. It could be possible that there are plant extracts that are well-tolerated, have selective toxicity for cancer cells, and target multiple vulnerabilities of cancer cells. Our research into dandelion root, lemongrass, long pepper, and hibiscus extracts has shown that these NHPs have the potential to induce apoptosis selectively in cancer cells [14–17]. These extracts have also been used widely and traditionally as food or medicinal products [18]. Thus, these extracts are well-tolerated and have the potential for long-term consumption to help prevent cancer remission. Although we have conducted research on DRE and LGE on colon cancer, leukemia, and lymphoma with high levels of success, their specific effect on prostate cancer is yet to be investigated. Furthermore, the interaction of DRE and LGE with standard cancer treatments has not yet been studied.

Dandelion (*Taraxacum officinale*) root has traditionally been used in Chinese, Arabian, and Native American traditional medicine for the purpose of treating diseases including digestive ailments to cancers [19]. Lemongrass (*Cymbopogon citratus*) has also been used traditionally and has shown anti-inflammatory, antimicrobial, and radical scavenging antioxidant properties in many diseases [20–22]. As mentioned previously, both of these extracts have been investigated in colon cancer, leukemia, and lymphoma cells, but their effect on prostate cancer remains undetermined [14, 15, 17, 23]. Our objective was to investigate DRE and LGE to assess their ability to induce apoptosis in prostate cancer cells* in vitro* and* in vivo*. More importantly, we wanted to assess the interaction of DRE and LGE with current chemotherapies to assess their potential use in adjuvant therapies.

In this study, we have shown that aqueous DRE and ethanolic LGE are able to induce apoptosis in prostate cancer in a dose- and time-dependent manner. Further, we have demonstrated that both treatments are selective for prostate cancer with no significant effect on normal healthy cells. Importantly, we have shown for the first time that DRE and LGE display positive interactions with current chemotherapeutic agents (i.e., they are able to enhance the efficacy of chemotherapies) on prostate cancer. The efficacy of DRE and LGE were further demonstrated in prostate cancer xenografts in immunocompromised mice, showing a reduction of tumour burden by oral administration of DRE and LGE. These results support the potential of DRE and LGE as viable alternatives to current treatments, as well as their validity in adjuvant therapies. This in turn may lead to the development of a well-tolerated long-term treatment option.

## 2. Materials and Methods

### 2.1. Dandelion Root Aqueous Extraction and Lemongrass Ethanolic Extraction

Asian dandelion (*Taraxacum officinale*) roots were obtained from Premier Herbal Inc. (Toronto, ON, Canada). The flowers were ground using a coffee grinder into a fine powder. The powder was extracted in boiled double distilled water (ddH_2_O) (1 g leaf powder to 10 mL ddH_2_O) at 60°C for 3 hours. The extract was then run through a cheese cloth and then filtered via gravity filtration with a P8 coarse filter, followed by vacuum filtration with a 0.45 *μ*m filter (PALL Life Sciences, VWR, Mississauga, ON, Cat No. 28148-028). The water extract was frozen at −80°C, freeze-dried using a lyophilizer, and then reconstituted in ddH_2_O in order to obtain a final stock concentration of 100 mg/mL. Prior to use, the water extract was passed through a 0.22 *μ*m filter (Sarstedt, Montreal, QC, Canada, Cat No. 83.1826.001) in a biosafety cabinet.

Lemongrass (*Cymbopogon citratus*) was obtained from Premier Herbal Inc. The lemongrass was ground using a coffee grinder into a fine powder. The powder was extracted in 100% anhydrous ethanol (1 g leaf powder to 10 mL anhydrous ethanol) at room temperature overnight. The extract was filtered via gravity filtration with a P8 coarse filter, followed by vacuum filtration with a 0.45 *μ*m filter. The extract was evaporated using a rotovap at 40°C and reconstituted in ethanol to obtain a final stock concentration of 200 mg/ml. The ethanolic extract was then passed through an Acrodisc® 0.2*μ*m DMSO-safe syringe filter in a biosafety cabinet.

### 2.2. Cell Culture

The prostate cancer cell line DU-145 (ATCC® HTB-81™) was cultured in Eagle's Minimum Essential Medium (EMEM) (ATCC® 30-2003™) supplemented with 10% (v/v) fetal bovine serum (FBS, Catalog No. 12484-020, Thermo Scientific, Waltham, MA, USA) and 0.4% (v/v) gentamicin (Catalog No. 15710-064, Gibco BRL, VWR, Mississauga, ON, CA).

The prostate cancer cell line PC-3 (ATCC® CRL-1435™) was cultured in F-12K Medium (Kaighn's Modification of Ham's F-12 Medium) (ATCC® 30-2004™) supplemented with 10% (v/v) fetal bovine serum (FBS) and 0.4% (v/v) gentamicin.

The normal colon mucosa cell line (ATCC® CRL-1831™) was cultured in Dulbecco's Modified Eagle's Medium (DMEM) (ATCC® 30-2002™) supplemented with 10% (v/v) fetal bovine serum (FBS) and 0.4% (v/v) gentamicin.

All cells were maintained in an incubator at 37°C with 5% CO_2_ and 95% humidity. All cells were passaged for less than 6 months.

### 2.3. Trypan Blue Exclusion Assay

Equal amounts of cells were seeded onto a 6-well clear bottom tissue culture plate and treated with various concentrations of aqueous DRE, ethanolic LGE, and chemotherapeutics. At 48 and 96 hours posttreatment, cells were collected and washed with phosphate buffer saline (PBS). Cells were then resuspended in 100 *μ*L of PBS and an equal amount (100*μ*L) of Trypan Blue solution (Catalog No. T8154, Sigma-Aldrich, Mississauga, ON, CA). The viable cells were counted using the Countess® II FL automated cell counter (Life Technologies, Burlington, ON, CA). Results were analyzed using the GraphPad Prism 6 software and expressed as “number of viable cells/mL” and expressed over a growth curve of the number of viable cells over the treatment period.

### 2.4. Analysis of Cell Death: Annexin V (AV) Binding Assay and Propidium Iodide (PI)

Annexin V binding assay and propidium iodide staining were performed to, respectively, monitor early apoptosis and cell permeabilization, a marker of necrotic or late apoptotic cell death. This assay is standardized, and we have used this assay in previous studies to analyze the effect of NHPs on various cancer cells [15, 24]. Cells were washed with phosphate buffer saline (PBS) and suspended in Annexin V binding buffer (10 mM HEPES, 140 mM NaCl, 2.5 mM CaCl_2_, pH 7.4) with green fluorescent Annexin V Alexa Fluor 488 (1:20) (Life Technologies Inc., Cat. No. A13201, Burlington, ON, Canada) and 0.01 mg/mL of red fluorescent PI (Life Technologies Inc., Cat. No. P3566, Burlington, ON, Canada) for 15 minutes at 37°C protected from light. Percentages of early (green) and late apoptotic cells (green and red) and necrotic cells (red) were quantified with a Tali Image-Based Cytometer (Life Technologies Inc., Cat. No. T10796, Burlington, ON, Canada). Cells from at least 18 random fields were analyzed using both the green (ex. 458 nm; em. 525/20 nm) and red (ex. 530 nm; em. 585 nm) channels. Fluorescent micrographs were taken at 400x magnification using LAS AF6000 software with a Leica DMI6000 fluorescent microscope (Wetzlar, Germany). Cells monitored with microscopy were counterstained with Hoechst 33342 (Molecular Probes, Eugene, OR, USA) with a final concentration of 10 *μ*M during the 15-minute incubation.

### 2.5. Reactive Oxygen Species (ROS) Scavenging

To assess the dependence of apoptotic induction on oxidative stress, the ROS scavenger N-acetylcysteine (NAC) was used to rescue cells. This assay is standardized, and we have used this assay in previous studies to analyze the effect of NHPs on various cancer cells [15, 24]. Cells were pretreated with 10 *μ*L NAC (Sigma-Aldrich Canada, Cat. No. A7250, Mississauga, ON, Canada) for 30 minutes at 37°C at 5% CO_2_. Cells were then treated for the indicated durations, collected, washed with phosphate buffer saline (PBS), and suspended in Annexin V binding buffer with green fluorescent Annexin V Alexa Fluor 488 (1:20) and 0.01 mg/mL of red fluorescent PI for 15 minutes at 37°C protected from light. Percentages of early (green) and late apoptotic cells (green and red) and necrotic cells (red) were quantified with a Tali Image-Based Cytometer. Cells from at least 18 random fields were analyzed using both the green (ex. 458 nm; em. 525/20 nm) and red (ex. 530 nm; em. 585 nm) channels.

### 2.6. Caspase Inhibition

To assess the caspase dependence of apoptotic induction, the caspase inhibitor Z-VAD-FMK was used to rescue cells. This assay is standardized, and we have used this assay in previous studies to analyze the effect of NHPs on various cancer cells [15, 24]. Cells were pretreated with 4 *μ*L Z-VAD-FMK (Sigma-Aldrich Canada, Cat. No. V116, Mississauga, ON, Canada) for 30 minutes at 37°C at 5% CO_2_. Cells were then treated for the indicated durations, collected, washed with phosphate buffer saline (PBS), and suspended in Annexin V binding buffer with green fluorescent Annexin V Alexa Fluor 488 (1:20) and 0.01 mg/mL of red fluorescent PI for 15 minutes at 37°C protected from light. Percentages of early (green) and late apoptotic cells (green and red) and necrotic cells (red) were quantified with a Tali Image-Based Cytometer. Cells from at least 18 random fields were analyzed using both the green (ex. 458 nm; em. 525/20 nm) and red (ex. 530 nm; em. 585 nm) channels.

### 2.7. *In Vivo* Assessment of the Dandelion Root and Lemongrass Extract Efficacy

Immunocompromised CD1 nu/nu mice, aged six weeks old, were obtained from Charles River Laboratories. Mice were housed and the protocols were followed using relevant guidelines and regulations that were approved by the University of Windsor Animal Care Committee (AUPP #17-15) in accordance with the Canadian Animal Care Committee in a laboratory setting with 12-hour light/dark cycles. Following an acclimatization period, mice were injected subcutaneously with prostate cancer cells (DU-145, PC-3); cells were suspended in Matrigel® at a concentration of 1.0x10^6^ cells per mouse in the hind flanks. Upon tumour formation, mice were randomly separated into three groups (control, DRE drinking water, and LG drinking water). Control and chemotherapeutic mice were given normal water, while DRE treatment groups received 40 mg/kg/day and LGE treatment groups received 16 mg/kg/day for 8 weeks. Mice were then sacrificed using CO_2_ chamber, followed by cervical dislocation, and tumours were harvested. Tumour volumes (using the formula 1/2*∗*(L*∗*W^2^) to calculate approximate volume) and body weights of each mouse were measured every other day throughout the length of the study.

### 2.8. Statistical Analysis

All statistical analyses were done using the GraphPad 6.0 Prism software. To test for statistical significance, a Two-Way Analysis of Variance (ANOVA) was used. All trials were conducted at least three independent times.

## 3. Result Text

### 3.1. Dandelion Root and Lemongrass Extracts Induce Apoptosis in a Dose- and Time-Dependent Manner in Prostate Cancer Cells

Hot water extract of dandelion root and ethanolic extract of lemongrass were prepared as described in Materials and Methods. To investigate the anticancer efficacy of DRE and LGE, prostate cancer cells were stained using Trypan Blue dye to assess cell permeability via image-based cytometry following 48- and 96-hour treatments. Prostate cancer cells (DU-145, PC-3) were additionally fluorescently stained with the apoptotic markers Annexin V (AV) and propidium iodide (PI). The cells were then subjected to fluorescent image-based cytometry following 48- and 96-hour treatments.

**Figure 1:**
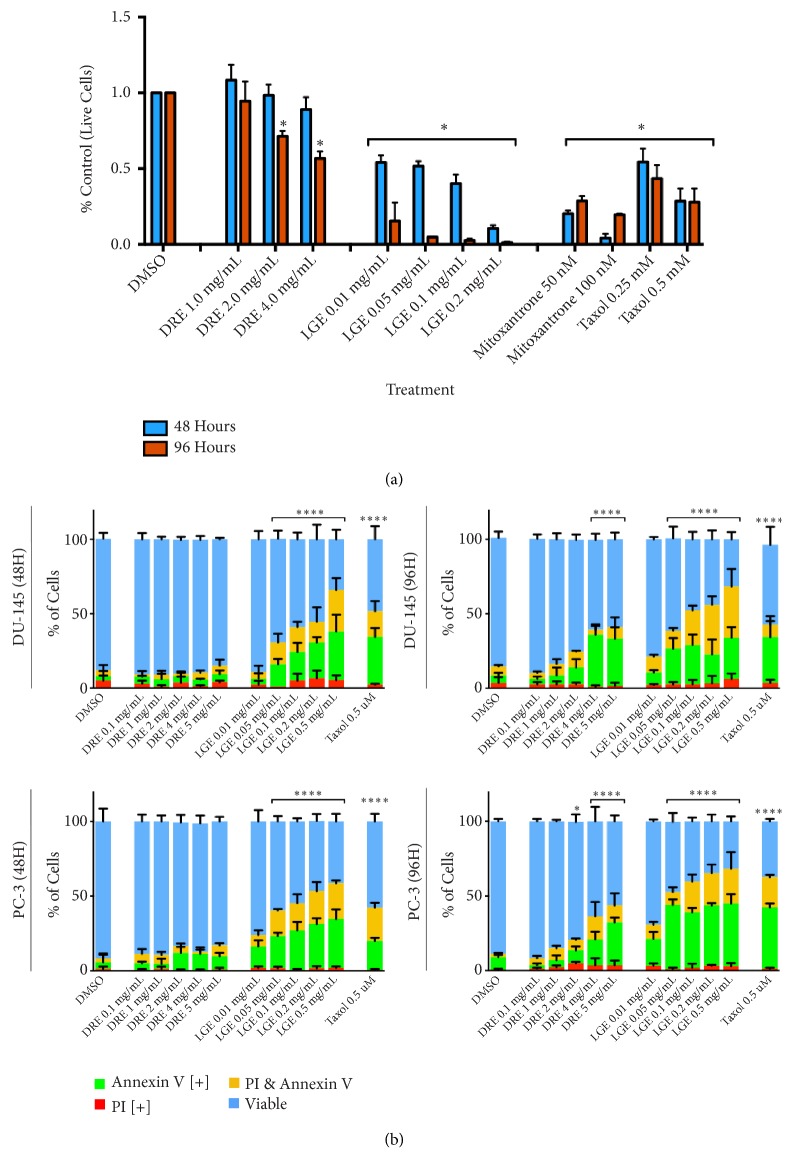
*Dandelion root and lemongrass extracts induce apoptosis in prostate cancer cells*. (a) Prostate cell line DU-145 was treated with various treatments of DRE, LGE, and chemotherapeutics taxol and mitoxantrone and assessed at 48 hours and 96 hours. Results were obtained using image-based cytometry to assess the percentage of live cells compared to a vehicle control. (b) Prostate cell lines DU-145 (top panels) and PC-3 (bottom panels) were treated with various treatments of DRE, LGE, and chemotherapeutics and assessed at 48 hours and 96 hours. Results were obtained using image-based cytometry to assess the percentage of cells positive with fluorescence associated with Annexin V (green), propidium iodide (PI, red), both (yellow), or negative for both Annexin V and PI (blue). Values are expressed as a mean ± SD from three independent experiments. Statistical calculations were performed using Two-Way ANOVA multiple comparison. **∗***p *< 0.05 vs. control, *∗∗p *< 0.01 vs. control, *∗∗∗∗p *< 0.0001 vs. control.

LGE was observed to be effective at inducing cell death through the Trypan Exclusion assay while DRE showed efficacy only at 96-hour treatment (Figure 1(a)). These observations were corroborated by fluorescence image cytometry results indicating that LGE and DRE are effective in inducing apoptosis as early as 48 hours and 96 hours posttreatment, respectively (Figure 1(b)). Specifically, significant apoptosis was observed in both prostate cancer cell lines for LGE beginning at dosages of 0.05 mg/mL (0.05 mg of crude extract in 1 mL of DMSO) at both timepoints. For DRE, significant apoptosis induction was observed beginning at 4 mg/mL treatments. Dosage- and time-dependent apoptosis induction was further observed in both cell lines as increased treatment concentration and treatment times increased apoptotic induction.

Both prostate cancer cell lines were further treated with taxol in order to compare the induction of apoptosis between standard chemotherapeutic treatment of taxol and DRE/LGE treatment. In both cell lines, DRE and LGE treatments resulted in comparable or enhanced apoptotic induction when compared to taxol treatment (Figure 1(b)).

### 3.2. Dandelion Root Extract and Lemongrass Extract Are Selective for Prostate Cancer Cells

**Figure 2:**
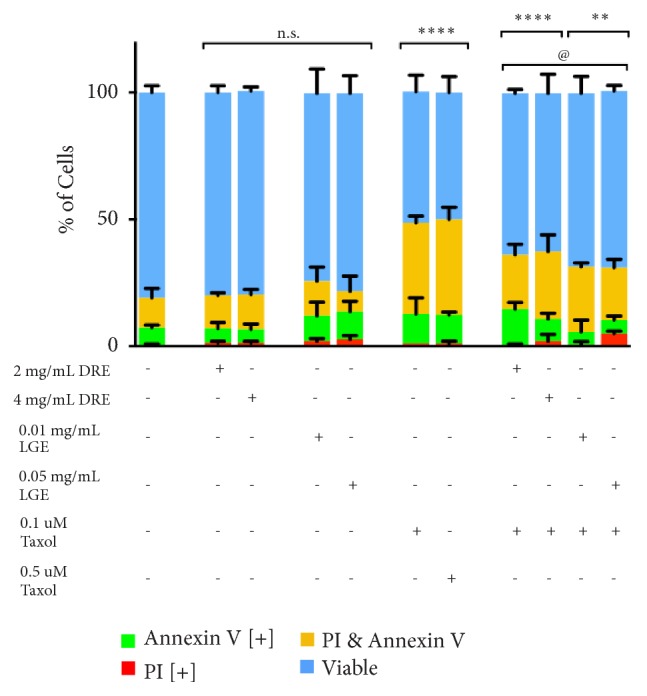
*Dandelion root and lemongrass extracts are selective for cancer and reduce toxicity of chemotherapeutics*. Normal colon mucosa (NCM) cells were treated with various dosages of DRE, LGE, and DRE or LGE combination treatments with taxol and assessed at 48 hours. Results were obtained using image-based cytometry to assess the percentage of cells positive with fluorescence associated with Annexin V (green), propidium iodide (PI, red), both (yellow), or negative for both Annexin V and PI (blue). Values are expressed as a mean ± SD from three independent experiments. Statistical calculations were performed using Two-Way ANOVA multiple comparison. *∗p *< 0.05 vs. control, *∗∗p *< 0.01 vs. control, *∗∗∗∗p *< 0.0001 vs. control, @p<0.05 vs. individual chemotherapy treatment.

If DRE and LGE treatments are selective for prostate cancer, the risk of drug-related side effects could be minimized. To assess whether DRE and LGE affect normal healthy noncancerous cells, normal colon mucosa (NCM) cells were treated with DRE and LGE and assessed using fluorescent image-based cytometry as described above. Compared to an untreated control, DRE and LGE treatments showed no significant increase in apoptotic induction (Figure 2). The highest dosages used in this experiment (4 mg/mL DRE, 0.05 mg/mL LGE) were able to induce significant apoptosis when used to treat prostate cancer cells, but not NCM cells. Further, while DRE and LGE treatments showed no effect on normal healthy cells, taxol induced apoptosis in these cells in a similar manner to that in prostate cancer cells.

To further investigate the benefit of combinatorial treatments of DRE and LGE, the effect of DRE or LGE addition to chemotherapy treatment on NCM cells was investigated. If protective, DRE or LGE would be able to minimize the amount of apoptotic induction in normal healthy cells by taxol. Indeed, we have observed that the addition of DRE and LGE to standard taxol chemotherapeutic treatment reduced the induction of apoptosis when compared to taxol treatment alone (Figure 2). These results indicate that DRE and LGE are selective for prostate cancer and can potentially protect normal healthy cells from being targeted by chemotherapy.

### 3.3. Interactions of Dandelion Root and Lemongrass Extracts with Standard Chemotherapies Taxol and Mitoxantrone

**Figure 3:**
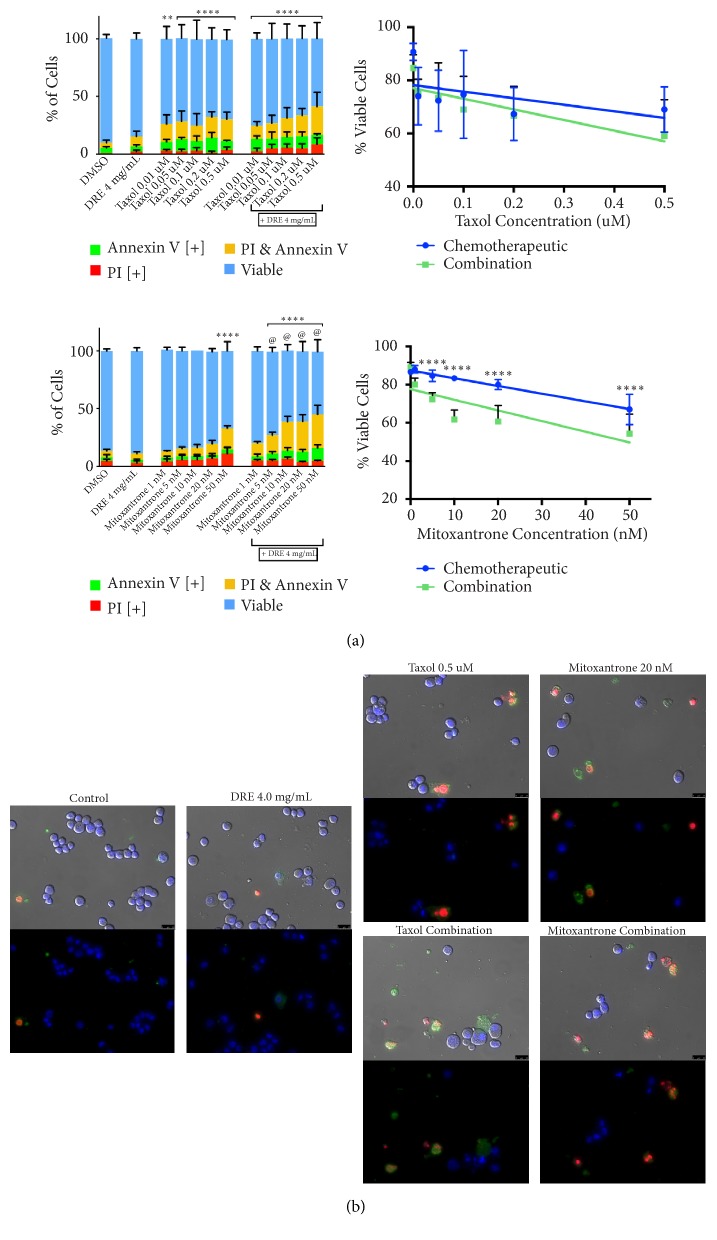
*Combination treatment with dandelion root extract enhances chemotherapeutic anticancer efficacy in prostate cancer cells*. (a) DU-145 cells were treated with chemotherapeutics taxol (top panel) and mitoxantrone (bottom panel) individually and in combination with 4 mg/mL DRE and assessed at 48 hours. Results were obtained using image-based cytometry to assess the percentage of cells positive with fluorescence associated with Annexin V (green), propidium iodide (PI, red), both (yellow), or negative for both Annexin V and PI (blue). Values are expressed as a mean ± SD from three independent experiments. The percentage of viable cells was graphed for both individual chemotherapeutic and combination chemotherapeutic treatments (graphs on right). (b) Fluorescence microscopy images of individual and hibiscus combination chemotherapeutic treatments. Top panels: brightfield and fluorescent merged images at 400x magnification. Bottom: fluorescent images stained with Annexin V (green), PI (red), and Hoechst (blue) at 400× magnification. Scale bar is 50 microns. Images are representative of three independent experiments. Statistical calculations were performed using Two-Way ANOVA multiple comparison. *∗p *< 0.05 vs. control, *∗∗p *< 0.01 vs. control, *∗∗∗∗p *< 0.0001 vs. control, @p<0.05 vs. individual chemotherapy treatment.

**Figure 4:**
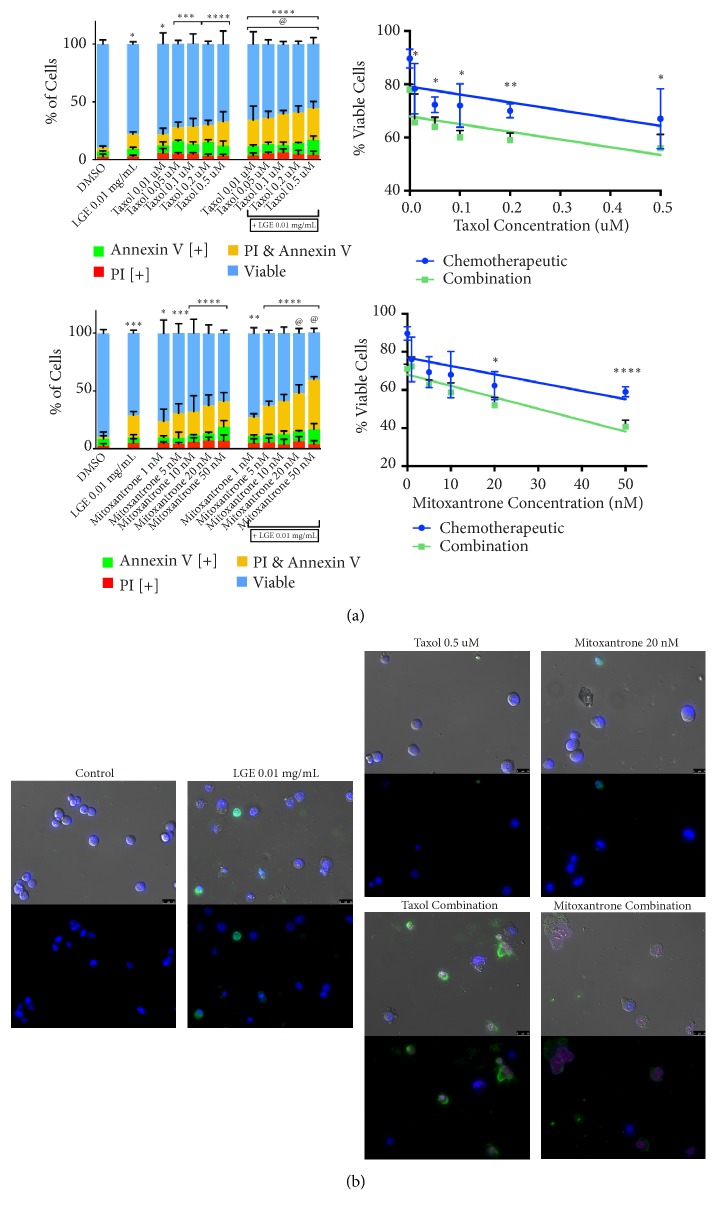
*Combination treatment with lemongrass extract enhances chemotherapeutic anticancer efficacy in prostate cancer cells*. (a) DU-145 cells were treated with chemotherapeutics taxol (top panel) and mitoxantrone (bottom panel) individually and in combination with 0.01 mg/mL LGE and assessed at 48 hours. Results were obtained using image-based cytometry to assess the percentage of cells positive with fluorescence associated with Annexin V (green), propidium iodide (PI, red), both (yellow), or negative for both Annexin V and PI (blue). Values are expressed as a mean ± SD from three independent experiments. The percentage of viable cells was graphed for both individual chemotherapeutic and combination chemotherapeutic treatments (graphs on right). (b) Fluorescence microscopy images of individual and hibiscus combination chemotherapeutic treatments. Top panels: brightfield and fluorescent merged images at 400x magnification. Bottom: fluorescent images stained with Annexin V (green), PI (red), and Hoechst (blue) at 400× magnification. Scale bar is 50 microns. Images are representative of three independent experiments. Statistical calculations were performed using Two-Way ANOVA multiple comparison. *∗p *< 0.05 vs. control, *∗∗p *< 0.01 vs. control, *∗∗∗∗p *< 0.0001 vs. control, @p<0.05 vs. individual chemotherapy treatment.

In standard treatments today, many chemotherapies are utilized in conjunction with other drugs. To properly assess the potential of DRE and LGE to be used in adjuvant or combination therapies in a novel treatment regimen, combination treatment assays were conducted to determine the interactions between DRE or LGE with taxol and mitoxantrone. These NHPs may either enhance, inhibit, or not affect chemotherapy efficacy. DU-145 prostate cancer cells were treated with taxol or mitoxantrone in the presence or absence of 4 mg/mL DRE (Figure 3) or 0.01 mg/mL LGE (Figure 4). As described in Materials and Methods, both image-based cytometry and fluorescence microscopy were used to analyze the ability of these treatments to induce apoptosis in prostate cancer cells.

The addition of 4 mg/mL DRE to mitoxantrone treatments was able to significantly increase the induction of apoptosis when compared to mitoxantrone treatment alone (Figure 3(a)). However, DRE did not seem to have any interaction with taxol and did not inhibit its action. The addition of 0.01 mg/mL LGE to both taxol and mitoxantrone treatments was able to significantly increase apoptosis induction compared to individual treatments (Figure 4(a)). Interestingly, the lowest combination dosage of taxol treatment (0.01 *μ*M with 0.01 mg/mL LGE) showed comparable induction of apoptosis to the highest individual treatment dosage of taxol (0.5 *μ*M). This indicates that LGE combination treatment was able to show a similar apoptosis induction to individual treatment with a 50-fold decrease in chemotherapeutic concentration.

Morphological assays were conducted to assess the effect of treatments on cell morphology. Fluorescent microscopy following Annexin V and propidium iodide staining after combination DRE treatments (Figure 3(b)) and combination LGE treatments at 48 hours (Figure 4(b)) confirmed apoptosis induction as described above. These apoptotic markers were observed in treated prostate cancer cells as expected, along with apoptotic morphology including cell shrinkage, membrane blebbing, and nuclear condensation.

### 3.4. Dandelion Root Extract-Induced Apoptosis Is Caspase Dependent, Whereas Lemongrass Extract-Induced Apoptosis Is Dependent on Oxidative Stress

**Figure 5:**
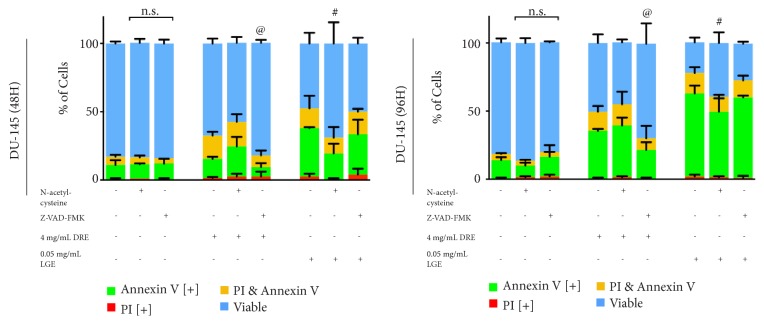
*Dandelion root extract apoptosis induction is caspase dependent and lemongrass extract apoptotic induction is oxidative stress dependent*. DU-145 cells were pretreated with either N-acetylcysteine (NAC, reactive oxygen species scavenger) or Z-VAD-FMK (caspase inhibitor), treated with various dosages of DRE, LGE, and DRE or LGE combination treatments with taxol, and assessed at 48 and 96 hours. Results were obtained using image-based cytometry to assess the percentage of cells positive with fluorescence associated with Annexin V (green), propidium iodide (PI, red), both (yellow), or negative for both Annexin V and PI (blue). Values are expressed as a mean ± SD from three independent experiments. Statistical calculations were performed using Two-Way ANOVA multiple comparison. *∗p *< 0.05 vs. control, *∗∗p *< 0.01 vs. control, *∗∗∗∗p *< 0.0001 vs. control, #p<0.05 vs. individual DRE treatment, @p<0.05 vs. individual LGE treatment.

DRE and LGE are extracts composed of a wide variety of compounds that can interact and function in complex manners. In order to further understand how these complex extracts exhibit their anticancer effects, the mechanism of apoptotic induction should be investigated. In order to determine if apoptosis is induced through oxidative stress, N-acetylcysteine (NAC) is used. NAC is a reactive oxygen species (ROS) scavenger and thus is able to rescue cells from apoptosis if the induction is primarily due to oxidative stress. LGE treatments on prostate cancer cells along with NAC were able to significantly reduce the amount of apoptosis induction when compared to individual LGE treatment whereas DRE treatments appear unaffected (Figure 5).

Further, to determine if apoptotic induction is caspase dependent or independent, Z-VAD-FMK, a commonly used broad-range caspase inhibitor, is used. If dependent, Z-VAD-FMK would be able to rescue cells and indicate dependence as described above. Indeed, DRE treatments were rescued when treating with DRE and Z-VAD-FMK, showing significant reduction of apoptotic induction when compared to individual DRE treatment (Figure 5). LGE treatments did not respond to caspase inhibition.

### 3.5. Oral Administration of Dandelion Root and Lemongrass Extracts Reduces the Tumour Burden in Prostate Cancer Xenograft Models in Immunocompromised Mice

**Figure 6:**
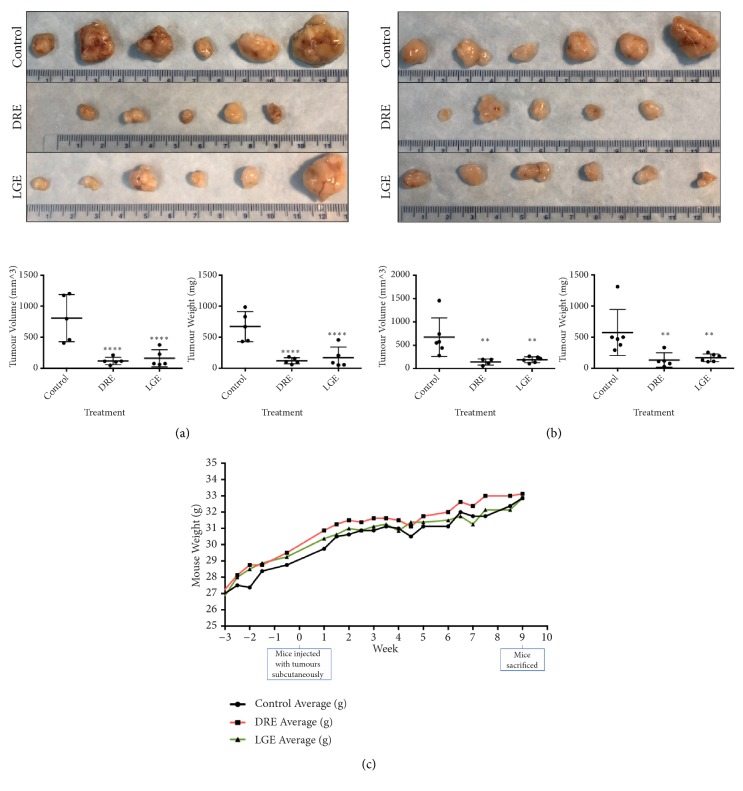
*Dandelion root and lemongrass extracts administered orally reduce the tumour burden on prostate cancer xenograft mice*. DU-145 (a) and PC-3 (b) prostate cancer cells were xenografted onto immunocompromised mice hind flanks subcutaneously. After tumour formation, these mice were orally fed dandelion root (DRE) and lemongrass (LGE) extract for 8 weeks. After mice were sacrificed, tumours (top panels) were excised and measured for tumour volume (using the formula 1/2*∗*(L*∗*W^2^) to calculate approximate volume) and tumour weight. (c) Mouse body mass was measured two times a week and averaged to compare between control, DRE, and LGE groups. Statistical calculations were performed using One-Way ANOVA multiple comparison. *∗*p < 0.05 vs. control, *∗∗*p < 0.01 vs. control, *∗∗∗∗*p < 0.0001 vs. control. No markings indicate nonsignificance vs. control.

To confirm and further support the anticancer efficacy of DRE and LGE, we wanted to investigate if these extracts have the ability to inhibit the growth of prostate cancer cells xenografted in immunocompromised mice. DU-145 and PC-3 cells were transplanted subcutaneously in mice. After tumour establishment, treatment groups were orally administered either DRE or LGE supplemented drinking water for eight weeks. Indeed, DRE and LGE were able to reduce the tumour burden of the xenografted mice as determined by tumour volume and weight compared to vehicle controls (Figures 6(a) and 6(b)). Over the course of treatment, there was no apparent change in the mice body weight gain in each treatment group when compared to controls, indicating that these treatments were well-tolerated (Figure 6(c)). Thus, DRE and LGE were effective and well-tolerated in reducing tumour burden and growth* in vivo* when administered orally.

## 4. Discussion

We have demonstrated in this report that DRE and LGE induce cell death in prostate cancer cells in a time and dosage dependent manner (Figure 1). In contrast to chemotherapeutics like taxol, apoptosis induction by these extracts is very selective for prostate cancer cells without any significant effect on normal colon mucosa (NCM) (Figure 2). Importantly, we have shown that adjuvant treatment with DRE and LGE in combination with taxol or mitoxantrone enhanced the apoptosis induction capacities of these drugs (Figures 3 and 4). Furthermore, we have evaluated the anticancer efficacy of both DRE and LGE* in vivo* where we have shown that these extracts were able to significantly and drastically reduce the tumour burden in mice with human prostate cancer xenografts. These extracts were administered orally, were well-tolerated, and did not affect mouse appetite or weight. These results support the potential of DRE and LGE supplementation in chemotherapeutic regimens due to observed enhancement of activity.

Previous work on DRE has shown its efficacy in inducing cell death in leukemia and colon cancer cells [14, 17]. However, the effects of DRE and LGE on human prostate cancer cells remained to be studied. Indeed, both DRE and LGE induced apoptosis in the two prostate cancer cell lines in a time- and dose-dependent manner. These cells displayed characteristic features of apoptosis including Annexin V binding. These extracts have been prepared using either water or ethanol; both are biocompatible solvents and thus fall in the category of food supplements. They have been traditionally consumed as teas or medicinal foods and have been shown to be well-tolerated. Although the dosages for DRE and LGE appear higher compared to pure compound treatments used in therapeutics, it is important to note that these are crude extracts of dandelion root and lemongrass. These extracts may contain many common compounds (salts and sugars) in addition to bioactive phytochemicals responsible for anticancer activity. We have previously shown through phytochemical analysis of extracts of dandelion root, lemongrass, and long pepper (*Piper longum*) that the active compound concentration is very low [14–16]. Further, we have shown that the active components of DRE and long pepper extract were ineffective in inducing apoptosis when used alone [16] indicating that these extracts may be effective through multiple compounds working together and targeting multiple pathways.

Generally, oncologists are cautious in advising the usage of natural health products (NHPs) alongside chemotherapies. This is primarily due to the potential of NHPs to interact negatively with chemotherapeutic treatment, reducing the efficacy of these drugs. Therefore, it is extremely important to investigate the interaction of these extracts with standard chemotherapeutics. We investigated these interactions between DRE, LGE, and common chemotherapeutics. Indeed, we have demonstrated that addition of 4 mg/mL DRE enhanced the efficacy of mitoxantrone while not significantly affecting taxol (Figure 3). Further, addition of 0.01 mg/mL LGE was able to enhance the efficacy of both taxol and mitoxantrone treatments (Figure 4). These results clearly indicate that the interactions that these NHPs have with chemotherapeutics are positive, enhancing the effect of chemotherapy. Most interestingly, addition of DRE to 1 nM mitoxantrone treatment indicated similar apoptotic induction at a concentration of 20 nM mitoxantrone alone. Similarly, addition of LGE to 0.01 *μ*M taxol resulted in effects similar to 0.5 *μ*M taxol. These results indicate that adjuvant therapy with DRE and LGE could potentially reduce the concentration required, thus avoiding drug-related adverse side effects. Thus, we have shown for the first time that these NHPs can be used as adjuvants to chemotherapies and potentially enhance their effect. Natural extracts have been used in patients on chemotherapies to enhance their quality of life in some cases [25].

The mechanism of apoptosis induction by these extracts in prostate cancer is a topic of great interest to determine the underlying cause of cell death. We have previously demonstrated that DRE and LGE were able to induce oxidative stress and decrease mitochondrial membrane potential, leading to apoptosis induction in cancer cells [14, 15]. Though the exact mechanism is not fully elucidated, it has been hypothesized that high reactive oxygen species (ROS) levels can activate cellular stress mechanisms and may sensitize cancer cells to further ROS production leading to apoptosis [7]. N-acetylcysteine (NAC), a known ROS scavenger, is able rescue cells by inhibiting apoptosis induction via oxidative stress [26]. Further, we can use the caspase inhibitor Z-VAD-FMK in order to assess the dependence of apoptosis induction on caspase action in a similar manner. This assay allows for the assessment of whether our treatments are ROS dependent or caspase dependent to induce apoptosis. Indeed, we have observed that LGE treatment along with NAC was able to rescue DU-145 cells (Figure 5) while LGE treatment with Z-VAD-FMK did not inhibit apoptosis, indicating that LGE treatment is ROS dependent and caspase independent. Interestingly, DRE treatment did not show any inhibition by NAC but was inhibited by Z-VAD-FMK, indicating that DRE treatment is caspase dependent and ROS independent. It is important to note that while these treatments showed some reduction, no cells were completely rescued, indicating that the mechanism of action of these extracts may be multifaceted and should be further investigated. Further work could look into quantifying the amount of ROS being produced via 2′, 7′-dichlorofluorescein diacetate (H_2_DCFDA) treatment or assessing different mechanisms of action to further assess these extracts.

As indicated previously, taxol and mitoxantrone have shown inadvertent side effects due to the nonselective targeting [9, 10, 27]. Indeed, we observed that taxol was toxic to normal colon mucosa (NCM) cells (Figure 2). We have shown in this study that LGE and DRE treatments were selectively toxic to prostate cancer cells without affecting normal noncancerous cells (Figure 2). Interestingly, addition of DRE or LGE to taxol was able to significantly reduce the extent of apoptosis in normal healthy cells compared to taxol treatment alone. This protective effect indicates the potential for DRE and LGE use in long-term adjuvant therapy in order to reduce the drug-related toxicity often associated with chemotherapy.

Some crude natural extracts have been shown to have anticancer efficacy against prostate cancer, but these studies were limited to* in vitro *work [28–30]. It is very important to assess the efficacy of natural extracts against prostate cancer using* in vivo *mice models with human prostate cancer xenografts. We carried out* in vivo* studies in which the mice (with human prostate cancer xenografts) were given DRE and LGE orally in their drinking water after tumour establishment. Indeed, both DRE and LGE were able to reduce the tumour burden of prostate cancer tumours significantly (Figures 6(a) and 6(b)) while having minimal effect on the overall weight gain of the mice (Figure 6(c)) indicating general tolerance. These results indicated that the anticancer bioactive compounds in these extracts must have been absorbed and stable upon systematic consumption and eventually affecting the tumour growth on subcutaneous site. This is important as we must consider that treatment was administered orally through mouse drinking water as opposed to methods such as intravenous or intraperitoneal administration. We have already demonstrated that both DRE and LGE are well-tolerated in mouse models [14, 15]. Thus, due to the fact that DRE and LGE treatment were efficacious against prostate cancer and well-tolerated, these NHPs are safe for long-term consumption prolonging the remission period.

## 5. Conclusions

In this study, we have demonstrated the efficacy of DRE and LGE for inducing cell death selectively in prostate cancer cells. For the first time, we have shown positive interactions of DRE and LGE with standard chemotherapeutics like taxol and mitoxantrone as they enhance their ability to induce apoptosis. Most importantly, addition of DRE and LGE led to reduced dosages of chemotherapies with the same extent of apoptosis compared to individual treatments (at high doses). Thus, DRE and LGE can reduce the drug-related toxicity with chemotherapeutics by reducing the dosage required for treatment. Further, oral administration of DRE and LGE to mice with human prostate cancer xenografts was able to significantly reduce the tumour burden. These extracts were well-tolerated in these mice as indicated by normal weight gain and food intake. Thus, these results suggest that DRE and LGE could potentially be used alongside mitoxantrone and taxol as adjuvants to enhance the efficacy of these drugs as well as improve the quality of life due to reduced toxicity for prostate cancer patients. These findings also provide support for the further development of these NHPs as a promising anticancer option for treating prostate cancer.

## Data Availability

The datasets used and/or analyzed during the current study are available from the corresponding author on reasonable request.
